# Green photodeposition of Au/TiO_2_ nanocomposites for wide-range photocatalytic degradation of pharmaceuticals

**DOI:** 10.1039/d6ra06617h

**Published:** 2026-10-14

**Authors:** Noelia González-Ballesteros, Inês Espada, Carlos J. Tavares, Senentxu Lanceros-Méndez, Pedro M. Martins

**Affiliations:** a Universidade de Vigo, Departamento de Química Inorgánica 36310 Vigo Spain noeliagb@uvigo.es; b Physics Centre of Minho and Porto Universities (CF-UM-UP) and LaPMET – Laboratory of Physics for Materials and Emergent Technologies, University of Minho 4710-057 Braga Portugal; c BCMaterials, Basque Centre for Materials, Applications and Nanostructures UPV/EHU Science Park 48940 Leioa Spain; d IKERBASQUE, Basque Foundation for Science 48009 Bilbao Spain; e Centre of Molecular and Environmental Biology (CBMA), University of Minho 4710-057 Braga Portugal; f Institute for Research and Innovation on Bio-Sustainability (IB-S), University of Minho 4710-057 Braga Portugal

## Abstract

Modifying photocatalytic materials by depositing noble metal nanoparticles is an effective strategy to enhance solar energy conversion. This study developed a UV-driven, one-pot photodeposition method to synthesise Au/TiO_2_ nanocomposites without chemical reducing agents. The influence of gold loading on physicochemical and photocatalytic properties was investigated. Structural, morphological, and optical analyses confirmed successful Au deposition without altering the TiO_2_ crystalline structure and also revealed improved colloidal stability and visible-light absorption due to Au surface plasmon resonance. The nanocomposites showed significant generation of reactive oxygen species under UV irradiation, with consistent hydroxyl radical production and increasing singlet oxygen formation at higher gold content. Photocatalytic performance was evaluated by degrading ciprofloxacin, chloroquine phosphate, diclofenac sodium, paracetamol, and fluconazole under UV light and simulated sunlight. A gold loading of 0.5 wt% achieved the highest efficiency, with up to 90% degradation of ciprofloxacin under both irradiation sources. Degradation of other pharmaceuticals ranged from 84% (chloroquine phosphate) to 45% (diclofenac sodium), depending on molecular structure and affinity for the catalyst. This work presents a simple and reproducible UV-driven approach to fabricating Au/TiO_2_ nanocomposites for the photocatalytic degradation of pharmaceutical pollutants.

## Abstract 2

A one-pot UV photodeposition method was developed to synthesise Au/TiO_2_ nanocomposites without reducing agents, enabling enhanced photocatalytic degradation of pharmaceutical pollutants.

## Graphical abstract

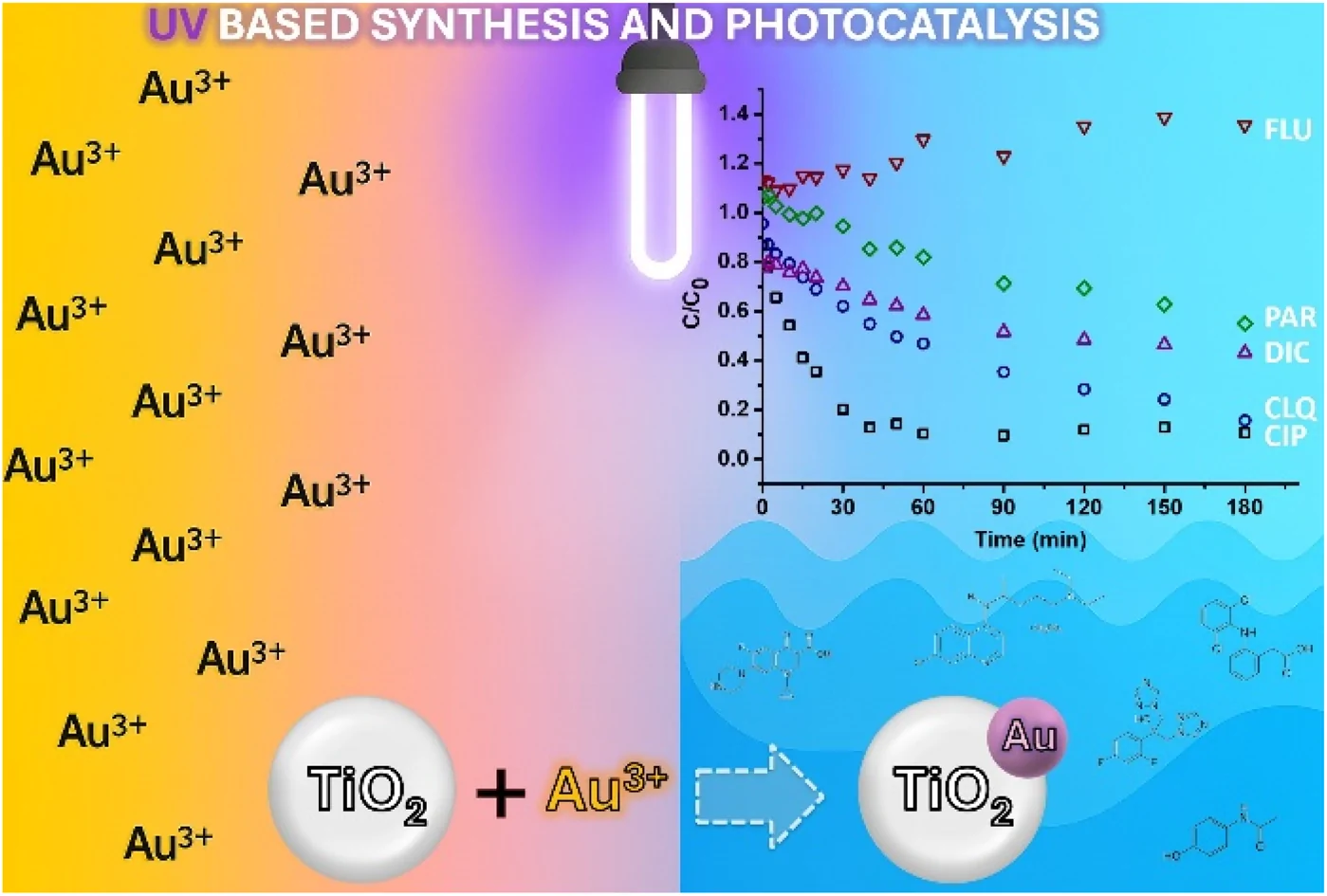

## 1 Introduction

The increased production and consumption of pharmaceuticals is directly related to advances in medicine and population growth, with a projected 12% rise in defined daily doses by 2028. ^1^ Among the most prescribed pharmaceuticals are antibiotics, antimalarials, analgesics, anti-inflammatory and antifungal pharmaceuticals, which include, respectively, ciprofloxacin (CIP), chloroquine phosphate (CLQ), diclofenac sodium (DIC), paracetamol (PAR) and fluconazole (FLU).^2^ Although they enhance the quality of life, their widespread use introduces serious health and environmental concerns. Pharmaceuticals are introduced to aquatic systems primarily through human and veterinary excretion, improper disposal of unused medications, agricultural runoff, and effluents from wastewater treatment plants that are not equipped to remove pollutants with such complex chemistries.^3^ These persistent contaminants of emerging concern (CECs) end up in surface or groundwater and, even at low concentrations (µg L^−1^), can have adverse ecological and health effects, such as disrupting the endocrine systems of aquatic organisms, promoting the development of antibiotic-resistant bacteria, and having long-term impacts on ecosystem dynamics.^4,5^

Photocatalysis has emerged as a promising, low-cost solution for efficiently degrading CECs among advanced oxidation processes. This simple technique relies on a photocatalyst's ability to produce electron–hole pairs when irradiated by an external radiation source (ultraviolet (UV) or visible) of energy equal to or higher than its semiconductor band gap. Both electrons and positive holes (h^+^) react with water molecules and oxygen to produce highly reactive oxygen species (ROS) that oxidise organic pollutants, consequently degrading them into harmless compounds.^6^

Titanium dioxide (TiO_2_) has been the most extensively researched photocatalyst in water remediation due to its low cost, abundance, unique optical properties, thermal and chemical stability, low toxicity and strong oxidative power.^7,8^ However, despite the demonstrated efficiency of UV-driven degradation of pharmaceuticals, its large-scale use is limited by a wide band gap (∼3.0 to 3.2 eV) that restricts absorption of the visible portion of the solar spectrum and by the rapid recombination of electron–hole pairs, which reduces photocatalytic efficiency.^9^

To overcome these limitations, TiO_2_ has been modified with a wide range of noble metal nanoparticles. Au, Ag and Pt, for example, can improve the separation and lifetime of photogenerated charge carriers by acting as electron sinks and forming metal–semiconductor interfaces. Au and Ag can further enhance the utilisation of visible light *via* surface plasmon resonance (SPR), while Pt has attracted considerable attention for its favourable electron-trapping properties and catalytic activity, which promote interfacial charge transfer and suppress electron–hole recombination.^10–12^ In parallel, non-noble metals, including Fe and Ni, have been investigated as more economically attractive alternatives for modifying TiO_2_ and extending its photocatalytic response.^13–15^ However, the performance and long-term stability of these systems can be strongly influenced by the oxidation state, metal-support interaction and susceptibility to metal leaching, which should be considered when developing photocatalysts for environmental applications. Carbon-based materials, such as reduced graphene oxide (rGO), have been considered more recently, with TiO_2_–rGO nanocomposites having been synthesised using *Neolamarckia cadamba* fruit extract, exhibiting efficient photocatalytic degradations of 91.9%, 85.8%, and 90.6% for ciprofloxacin, ibuprofen, and tetracycline hydrochloride under visible light.^16^ Although carbon-based functionalisation improve charge separation, poor interfacial stability and susceptibility to photo-oxidation lead to structural degradation during long-term operation. Therefore, among different metal and non-metal nanoparticles, Au is often preferred due to its superior resistance to long-term oxidation and the promising efficiencies reported for visible-induced photocatalytic degradation of pharmaceuticals.^17^

Although the photocatalytic efficiency of Au/TiO_2_ systems under solar irradiation has been widely investigated, the preparation route plays an important role in determining the physicochemical properties, environmental impact and potential scalability of the resulting photocatalyst. Conventional methods for the synthesis of metal-modified TiO_2_ commonly involve chemical reducing and stabilising agents, which may generate undesirable by-products and increase the environmental burden of the synthesis process.^18,19^ In recent years, greener approaches based on plant, algal, or other biological extracts have been proposed as alternatives to conventional chemical synthesis, leveraging naturally occurring reducing and capping compounds.^20–23^ Nevertheless, the complex and variable composition of natural extracts can influence metal-ion reduction, nucleation and nanoparticle growth, potentially affecting particle size, morphology, metal loading and, consequently, photocatalytic performance. Variations associated with the biological source, harvesting conditions and extraction procedure may therefore compromise batch-to-batch reproducibility and make the standardisation and scale-up of these approaches challenging.^24^

UV-mediated photodeposition has been successfully employed to prepare several metal/TiO_2_ systems with enhanced photocatalytic or antimicrobial properties. For example, Pt and Ag nanoparticles have been photodeposited onto TiO_2_ and evaluated for the degradation of pharmaceutical contaminants and antibacterial applications, respectively.^25,26^ Other studies have reported Au–Ag/TiO_2_ and TiO_2_/MoS_*x*_/Ag heterostructures prepared through light-assisted approaches for the degradation of model organic pollutants such as methylene blue and rhodamine B.^27,28^ These studies demonstrate the potential of photodeposition to improve the photocatalytic properties of TiO_2_; however, the reported applications have frequently focused on a single model contaminant or on dyes, while comparatively less attention has been given to the solar-driven degradation of structurally diverse pharmaceutical contaminants using Au/TiO_2_ prepared directly by UV photodeposition. Moreover, differences in pollutant structure, functional groups and light-absorbing properties can substantially influence their interaction with the photocatalyst and their susceptibility to oxidative degradation. A systematic evaluation using chemically diverse pharmaceuticals under comparable experimental conditions is therefore necessary to better understand the factors governing their photocatalytic degradation.

Therefore, this work introduces a sustainable one-pot UV-mediated photodeposition strategy for the direct preparation of Au nanoparticles on TiO_2_ without the addition of external chemical reducing or capping agents. The resulting Au/TiO_2_ photocatalysts were evaluated under simulated solar radiation for the degradation of five widely prescribed pharmaceuticals, namely ciprofloxacin (CIP), chloroquine phosphate (CLQ), diclofenac sodium (DIC), paracetamol (PAR) and fluconazole (FLU), selected because of their substantially different chemical structures and functional groups. Rather than focusing on a single model pollutant, this study aims to provide a comparative assessment of the photocatalytic response of Au/TiO_2_ toward structurally diverse pharmaceutical contaminants under comparable irradiation conditions. Particular attention is given to the influence of the photocatalyst's physicochemical properties, irradiation conditions, and pollutant-specific structural features on the degradation process, providing insights into the potential of UV-photodeposited Au/TiO_2_ for the solar-driven remediation of pharmaceutical contaminants.

## 2 Materials and methods

### 2.1 Reagents

TiO_2_ P25 (99.5% – 35–65 m^2^ g^−1^) was purchased from Evonik. Gold(iii) chloride solution (99.99%), chloroquine phosphate (CLQ, Pharmaceutical Secondary Standard), paracetamol (PAR, European Pharmacopoeia (EP) Reference Standard), and diclofenac sodium (DIC, European Pharmacopoeia (EP) Reference Standard) were supplied by Sigma-Aldrich. Ciprofloxacin (CIP, 98%) was acquired from Acros Organics, and Fluka Analytical provided Fluconazole (FLU, 98%). All reagents were used without further purification, and Milli-Q water was employed throughout all experiments.

### 2.2 Synthesis of Au/TiO_2_ nanocomposites under UV irradiation

The UV-mediated photodeposition method for synthesising Au/TiO_2_ nanocomposites is schematised in Fig. 1.

**Fig. 1:**
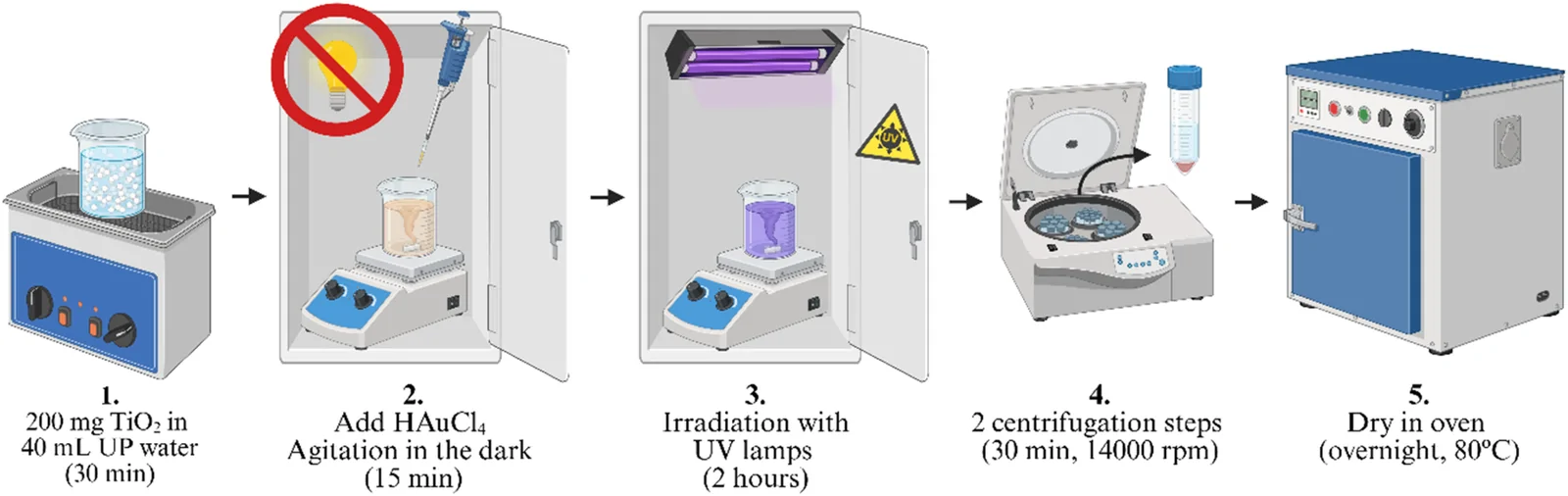
UV-mediated photodeposition synthesis of Au/TiO_2_ nanocomposites.

Firstly, 200 mg of TiO_2_ was dispersed in 40 mL of Milli-Q water in a sonication bath (ULTRASONS-HD, JP Selecta, Spain) for 30 min at room temperature. Then, different volumes of HAuCl_4_ (0.05 wt%) were slowly added under constant stirring to obtain Au loadings of 0.025%, 0.05%, 0.1, 0.5% and 1%, stirring for 15 min in the dark to achieve a homogeneous distribution of gold ions. Afterwards, the reaction mixture was irradiated with UV light under constant stirring using a homemade photoreactor chamber equipped with eight 8 W UV lamps (emission at 365 nm) and an irradiance of 3.3 W m^−2^. The distance between the reaction mixture and the lamp was 13.5 cm, with irradiation from the top. A colour change from pale yellow to purple was observed after 2 hours of reaction, confirming the formation of Au nanoparticles. Then, the samples were centrifuged (Sigma 4–16 KS, Sigma, Germany) at 14 000 rpm for 30 min and then redispersed in ultrapure water under ultrasound. This step was performed twice, and finally, the samples were dried at 80 °C in an oven overnight (Digitronic, JP Selecta, Spain).

### 2.3 Characterisation techniques

Transmission electron microscopy (TEM) images were obtained to characterise the morphology of the samples using a JEOL JEM-1010 (JEOL, Tokyo, Japan) operating at 100 kV. For sample preparation, Au/TiO_2_ nanocomposites were dispersed in ultrapure water and sonicated for 30 min. A drop of the nanoparticles’ dispersion was placed on 400-mesh Formvar/carbon-supported copper grids and dried at room temperature.

A JEOL JEM 2010F field-emission gun TEM (JEOL, Tokyo, Japan), operated at 200 kV, was employed for the acquisition of high-resolution (HRTEM) and scanning transmission (STEM) images of 0.5% Au/TiO_2_. Coupling between the STEM unit and the energy-dispersive X-ray (EDS) detector (Oxford Inca Energy 200, Oxford Instruments Analytical, High Wycombe, UK) was used to obtain EDS spectra and elemental maps. In all cases, data collection and analysis were carried out using DigitalMicrograph software from Gatan, and, for EDS, INCA Energy software was also used.

X-ray powder diffraction (XRD) analysis of TiO_2_ and Au/TiO_2_ nanocomposites was performed in a D8 Discover diffractometer from Bruker (Billerica, Massachusetts, USA). The incident Cu Kα operated at a voltage of 40 kV and a current of 30 mA. The 2*θ* range analysis was performed from 20° to 80° with a step size of 0.02°.

A Jasco FT/IR-6100 spectrophotometer (JASCO, Tokyo, Japan) was employed to acquire Fourier-transform infrared spectra using the potassium bromide pellet technique in the 4000–400 cm^−1^ range at a resolution of 4 cm^−1^.

Dynamic light scattering (DLS) and zeta potential (Z-potential) measurements were performed in a ZetasizerNano S (Malvern Instruments, Malvern, UK) equipped with a He–Ne laser (wavelength 633 nm) and backscatter detection (173°). Samples were prepared by dispersion of the dried powder in ultrapure water at a concentration of 0.5 mg mL^−1^ in an ultrasonic bath for 30 min to prevent aggregation. In the case of Z-potential, measurements were acquired at different pH levels (3, 5, 7, 9, and 11) adjusted with HCl and NaOH solutions. All measurements were performed 5 times. The software Zetasizer 7.13 was used to determine particle diameter (intensity distribution), polydispersity index (PDI), and Z-potential.

UV-Vis diffuse reflectance spectroscopy (DRS) analysis of the samples was performed using a Shimadzu UV-2501-PC (Shimadzu Corporation, Kyoto, Japan) and an integrating sphere. BaSO_4_ reference samples were employed to measure the 100% reflectance. DRS measurements were conducted in the wavelength range of 240–800 nm. The obtained reflectance spectra were converted to absorbance through the Kubelka–Munk (K–M) eqn (1).^29^

1 *F*(*R*) = (1 − *R*_∞_)^2^/(2*R*_∞_)

where *R*_∞_ = (*R*_Sample_/*R*_BaSO_4__) corresponds to the reflectance of the sample and *F*(*R*) is the absorbance.

The band gap (*E*_g_) values were estimated using Tauc plots (eqn (2)).

2 [*F*(*R*)*hυ*]^1/*n*^*versus hυ*

where *h* is the Planck constant (6.626 × 10^−34^ J s), *υ* is the frequency, and *n* is the sample transition (indirect transition, *n* = 2).

### 2.4 Photogenerated ROS measurements

#### 2.4.1 Detection of hydroxyl radicals (˙OH)

The hydroxyl radicals (˙OH) produced under UV irradiation were quantified by fluorescence spectroscopy using a method previously reported,^30^ based on the hydroxylation reaction of terephthalic acid (TA) to 2-hydroxyterephthalic acid (2-HTA) in the heterogeneous phase.

Briefly, 50 mg of the catalyst (Au/TiO_2_ nanocomposites) were dispersed in 50 mL of TA solution (0.5 mM, in NaOH 2 mM) for 30 min in the dark. Then, the suspension was irradiated with UV light for 60 min under constant stirring. Aliquots were taken at times 0, 2, 5, 10, 15, 20, 30, 40, 50, and 60 min. Samples were centrifuged at 14 000 rpm for 30 min to remove the photocatalyst, and the fluorescence of the supernatants was measured using a Tecan Infinite M Plex microplate reader, with an excitation wavelength of 315 nm and an emission wavelength of 425 nm. A calibration curve was composed using 2-hydroxyterephthalic acid at concentrations ranging from 0.001 to 0.01 mM.

#### 2.4.2 Detection of singlet oxygen (^1^O_2_)

Singlet oxygen (^1^O_2_) production under UV irradiation was quantified using the histidine method, as previously reported.^30^

Firstly, 50 mg of nanocomposites were dispersed in a solution containing 40 mL of l-histidine (0.2 mM) and 10 mL of *N*,*N-p*-nitrosodimethylaniline (0.2 mM) for 30 min in the dark. Afterwards, the suspension was irradiated with UV light for 60 min while stirring. Aliquots were withdrawn at different irradiation times (0, 2, 5, 10, 15, 20, 30, 40, 50 and 60 min) and centrifuged at 14 000 rpm for 30 min. Then, the absorbance of the supernatant was measured using a microplate reader (Tecan Infinite M Plex) between 350 and 550 nm. A calibration curve was composed using *N*,*N-p*-nitrosodimethylaniline at concentrations ranging from 0.05 to 0.0001 mM.

### 2.5 Photocatalytic degradation evaluation

All the synthesised samples were evaluated in two independent assays for the photocatalytic degradation of the antibiotic CIP under UV and simulated sunlight radiation.

CIP solution was prepared at a concentration of 5 mg L^−1^ in ultrapure water and adjusted to pH 3 using HCl to ensure the complete dissolution of the antibiotic. This concentration was selected based on previous reports and considering the limitations in detecting CIP using a UV-Vis spectrophotometer.^17^ UV degradation assays were performed using the same home-made photoreactor chamber as in the nanocomposite synthesis.

The visible light degradation experiments utilised a Xenon lamp (emulating the sun's radiation) with an excitation peak at 550 nm and an irradiance of 300 W m^−2^. The beaker containing the samples was placed 20 cm from the lamp under lateral illumination.

For all assays, 50 mg of catalyst was mixed with 50 mL of CIP at 5 mg L^−1^ in a 100 mL beaker and stirred in the dark for 30 min to reach adsorption/desorption equilibrium. Then, the samples were irradiated with UV or simulated sunlight radiation for 60 min and 180 min, respectively. 1.5 mL aliquots were collected at different times and centrifuged at 14 000 rpm for 30 min to separate the solution from the photocatalyst. UV-Vis spectra of the supernatant were obtained with the Tecan Infinite M Plex microplate reader, operating at room temperature in the 230–400 nm range. The effectiveness of the synthesised catalysts was evaluated by monitoring the absorbance of CIP (maximum at 277 nm) over time. The degradation rate of CIP was adjusted to the Langmuir–Hinshelwood model, fitting a pseudo-first-order reaction as a function of time (*t*) (eqn (3)).^17^

3 *C*/*C*_0_ = e^−*kt*^,

where *C*_0_ is the CIP concentration at *t* = 0, *C* is the CIP concentration at a given time and *k* is the first-order reaction rate constant.

All assays were performed in triplicate, and the results presented are the mean value of the replicates. The efficiency of pristine TiO_2_ nanoparticles in degrading CIP was also assessed for comparison.

The sample with the best degradation efficiency against CIP was then selected for the degradation of other pharmaceutical compounds under UV and simulated sunlight irradiation. The chosen compounds were CLQ, DIC, PAR and FLU. Table 1 summarises the chemical structure of each contaminant and their characteristic absorption peak. Their p*K*_a_ at pH 3 is also listed, as it provides information on the pharmaceutical's charge and form, which influences its surface affinity to the photocatalyst (adsorption), reactivity with ROS, and overall degradation efficiency.

**Table 1:** Chemical structure, characteristic absorption peak, p*K*_a_ and form at pH 3 of the pharmaceuticals CIP, CLQ, DIC, PAR and FLU

Contaminant	*λ*_max_	p*K*_a_	Form at pH 3	Ref.
CIP (C_17_H_18_FN_3_O_3_)	277 nm	6 (Carboxylic acid group)	Cationic	31
		8.7 (Piperazine nitrogen)		
CLQ (C_18_H_26_ClN_3_·H_3_PO_4_)	342 nm	8.5 to 10.1 (Quinoline nitrogen)	Dicationic	32
DIC (C_14_H_10_Cl_2_NO_2_Na)	276 nm	4 (Carboxylic acid group)	Neutral	33
PAR (C_8_H_9_NO_2_)	260 nm	9.5 (Phenolic hydroxyl group)	Neutral	34
FLU (C_13_H_12_F_2_N_6_O)	243 nm	2.6 and 2.9 (Triazole rings	Cationic	35
		11 (Hydroxyl group)		

The previous CIP protocol was applied to each compound, with adjusted pharmaceutical concentrations to monitor their degradation by UV-Vis spectroscopy. The concentrations employed were CLQ at 30 ppm, DIC at 30 ppm, PAR at 15 ppm, and FLU at 200 ppm. The photocatalytic efficiency was evaluated by monitoring the decrease in the main absorption peak (*λ*_max_) of each compound in the UV-Vis spectra of aliquots collected at different time points during the degradation assays.

## 3 Results and discussion

### 3.1 Synthesis and characterisation of Au/TiO_2_ nanocomposites

Au nanoparticles were successfully synthesised and deposited on the surface of TiO_2_*via* a one-pot approach without additional reagents. The colour changes from pale yellow to purple suggest that the Au(iii) precursor was reduced into Au(0). When TiO_2_ is irradiated with UV light, electrons (e^−^) are excited from the valence band to the conduction band, leaving positive charge holes (h^+^) behind. These electron–hole pairs, or charge carriers, can initiate reduction or oxidation reactions with chemical species deposited on the TiO_2_ surface, depending on the species' redox potential.

According to Butler *et al.*,^36^ the conduction band and valence band edge potentials of a semiconductor can be calculated following the equations:

4 *E*_CB_ = *χ* − *E*_e_ − 0.5*E*_g_

5 *E*_VB_ = *E*_CB_ + *E*_g_

where *χ* = 5.81 eV is the absolute electronegativity of TiO_2_,^37^*E*_e_ = 4.5 eV is the energy of a free electron on the hydrogen scale^38^ and *E*_g_ = 3.2 eV is the commonly reported band gap of anatase TiO_2_.^39^ This gives *E*_CB_ = −0.29 V and *E*_VB_ = +2.91 V corresponds to the redox potential of the excited electron and hole of TiO_2_.

Considering the initial solution of HAuCl_4_ (0.05 wt%), the following half reactions can be considered:

6 HAuCl_4_ → H^+^ + AuCl_4_^−^

7 AuCl_4_^−^ + 3e^−^ → Au^0^ + 4Cl^−^

The reported standard reduction potential of eqn (7) is *E*_0_ = +0.994 V ^40^. Assuming complete dissociation and knowing the theoretical concentrations [HAuCl_4_] = [AuCl_4_^−^] = 1.27 × 10^−3^ M and [Cl^−^] = 5.08 × 10^−3^ M, the simplified Nernst equation can be utilised to determine the redox potential ^41^:8
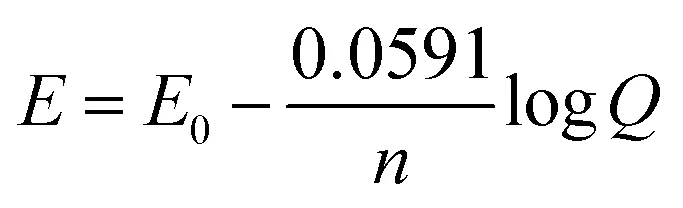
where *n* = 3 is the number of transferred electrons and *Q* = [Cl^−^]^4^/[AuCl_4_^−^] is the reaction quotient. This gives a AuCl_4_^−^/Au^0^ potential of *E* = +1.145 V. Since the excited electrons of TiO_2_ present a higher energy, the gold precursor can be successfully reduced under the experimental conditions considered. At the same time, holes generated in the valence band can oxidise other molecules in solution, such as water, thereby maintaining charge neutrality in the system.

Fig. 2 shows TEM images of pristine TiO_2_ and Au/TiO_2_ nanocomposites with different Au loadings. The TiO_2_ nanoparticles exhibit the morphology characteristic of the P25 material, with nanoparticles forming aggregates. No noticeable changes in the overall structure or morphology of the TiO_2_ nanoparticles were observed after Au incorporation. The Au nanoparticles have a size comparable to that of the TiO_2_ nanoparticles, and, together with their similar contrast in conventional TEM, this makes them difficult to distinguish clearly from the TiO_2_ support, particularly at lower Au loadings. Therefore, conventional TEM does not allow reliable determination of the size, shape, and distribution of the deposited Au nanoparticles, or direct comparison among different Au loadings. For this reason, STEM imaging with compositional contrast, in which the higher atomic number of Au yields brighter contrast than Ti, was employed for the 0.5 wt% Au/TiO_2_ sample (Fig. 3a). This distinction was further confirmed by EDX analysis (Fig. 3b) and elemental mapping (Fig. 3c–e), where gold is represented in red, titanium in blue, and oxygen in green. The STEM-EDX results confirmed the presence and distribution of Au over the TiO_2_ nanoparticles.

**Fig. 2:**
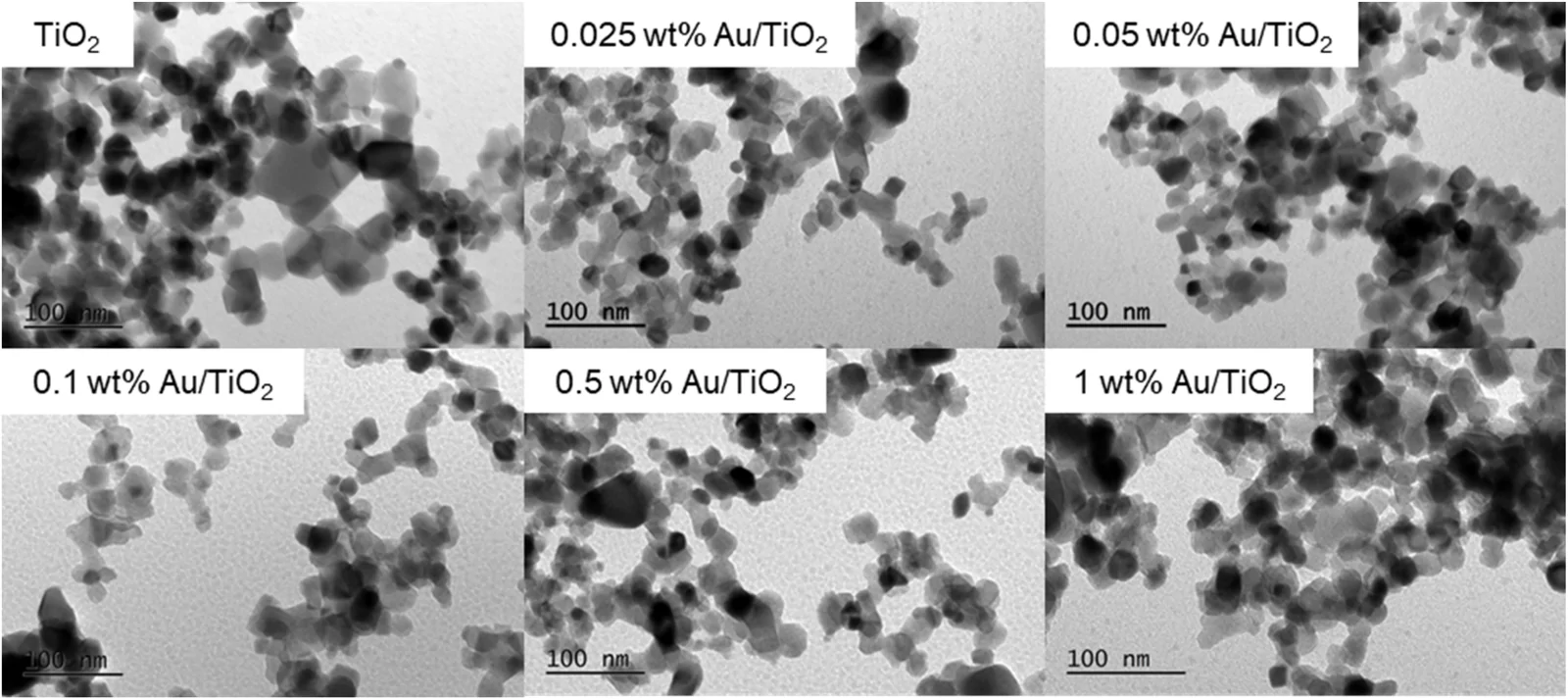
TEM images of pristine TiO_2_ before the synthesis and Au/TiO_2_ nanocomposites with different gold loadings.

**Fig. 3:**
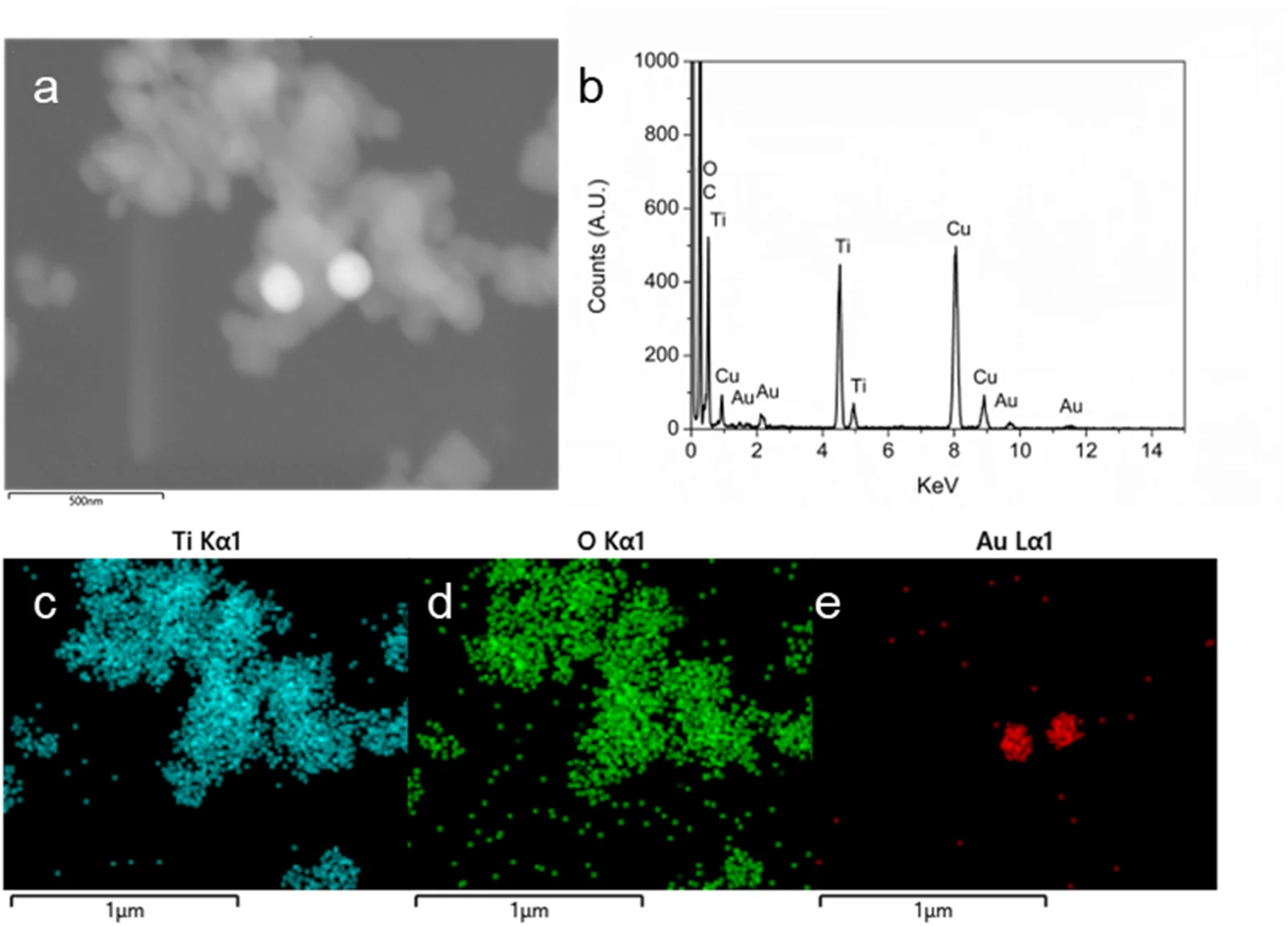
(a) STEM image of 0.5% Au/TiO_2_. (b) The EDX spectrum of the area is shown in (a). (c–e) Elemental mapping showing titanium, oxygen and gold distribution.

The crystallinity of the nanocomposites was assessed by XRD and compared with that of pristine TiO_2_, as shown in Fig. 4a. The XRD diffraction patterns reveal distinctive peaks at 25.27, 36.95, 37.82, 48.03, 53.97, 55.04, 62.73, and 68.89°, corresponding to the diffraction from (101), (103), (004), (200), (105), (211), (204), and (116) atomic planes of anatase, while peaks observed at 27.43, 36.94, and 37.81° are attributed to the diffraction from (110), (101), and (111) atomic planes of rutile, confirming the typical crystalline structure of pristine TiO_2_. ^42,43^ Notably, no discernible changes in the Au/TiO_2_ spectra are observed in the TiO_2_-associated peaks, indicating that UV light irradiation during synthesis did not affect the crystallinity. The expected diffraction peaks of gold (JCPDS file: 04-0784) should appear at 38.2°, 44.5°, and 64.7°, assigned to the diffraction from the (111), (200), and (220) planes of Au, respectively (marked with dotted lines in Fig. 4a). However, the most intense Au peak at 38.2° is masked by one of the anatase peaks (37.82°) due to the higher concentration and crystalline nature of TiO_2_.

**Fig. 4:**
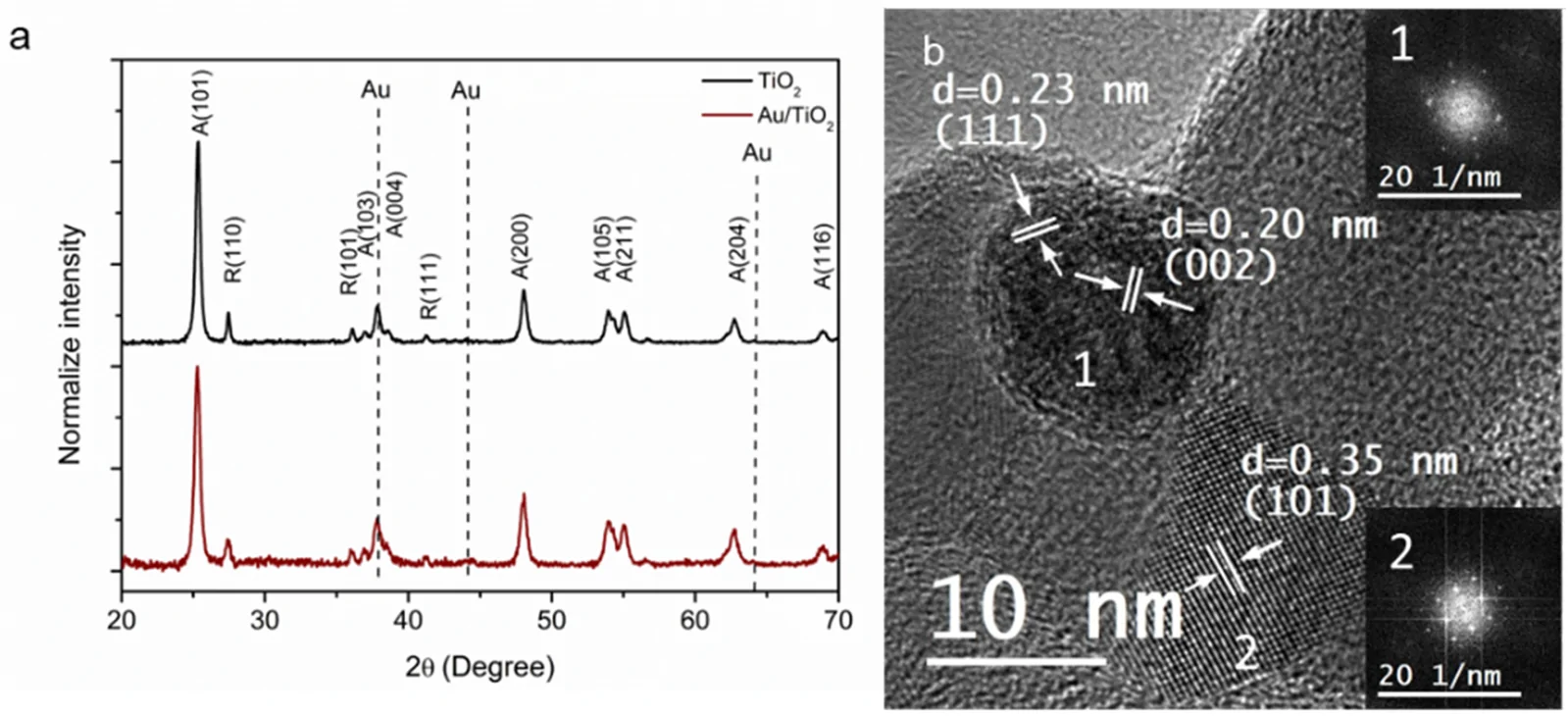
(a) XRD patterns of TiO_2_ and Au/TiO_2_. Dot lines represent peak positions expected for Au nanoparticles (JCPDS file: 04-0784). (b) HRTEM image of Au/TiO_2_. Inset corresponding to the FFT of the marked nanoparticles.

Therefore, the crystallinity of the Au nanoparticles was confirmed by HRTEM (Fig. 4b). In the image, two particles appear. Particle 1 is a polycrystalline nanoparticle formed by several small crystalline domains.^44^ The analysis of the fast Fourier transform (FFT) (inset Fig. 4b) and comparison with tabulated data allowed the calculation of the interplanar distances of the crystalline structure, obtaining values of 0.23 and 0.20 nm, which respectively correspond to the (111) and (002) atomic planes of the face-centred cubic (FCC) structure *Fm*3̄*m* (No. 225) of Au nanoparticles. Conversely, particle 2 is a monocrystalline nanoparticle with an interplanar distance of 0.35 nm assigned to the (101) atomic planes of anatase.^43^

Fig. 5a shows the FTIR spectra of pristine TiO_2_ and those of Au/TiO_2_ nanocomposites with varying Au loadings. All spectra show the characteristic bands of TiO_2_. A broad band at ∼3430 cm^−1^ appears, assigned to O–H stretching vibrations of hydroxyl groups and absorbed water moieties.^45^ This band loses intensity due to the presence of Au nanoparticles or to thermal drying, which reduces water adsorption and hydroxylation on the TiO_2_ surface.^46,47^ The small band appearing at ∼1640 cm^−1^ is assigned to the bending and stretching vibrations of Ti–OH.^48^ The broad, intense band between 1000 and 400 cm^−1^ has been attributed to Ti–O and Ti–O–Ti bridging stretching modes.^49^

**Fig. 5:**
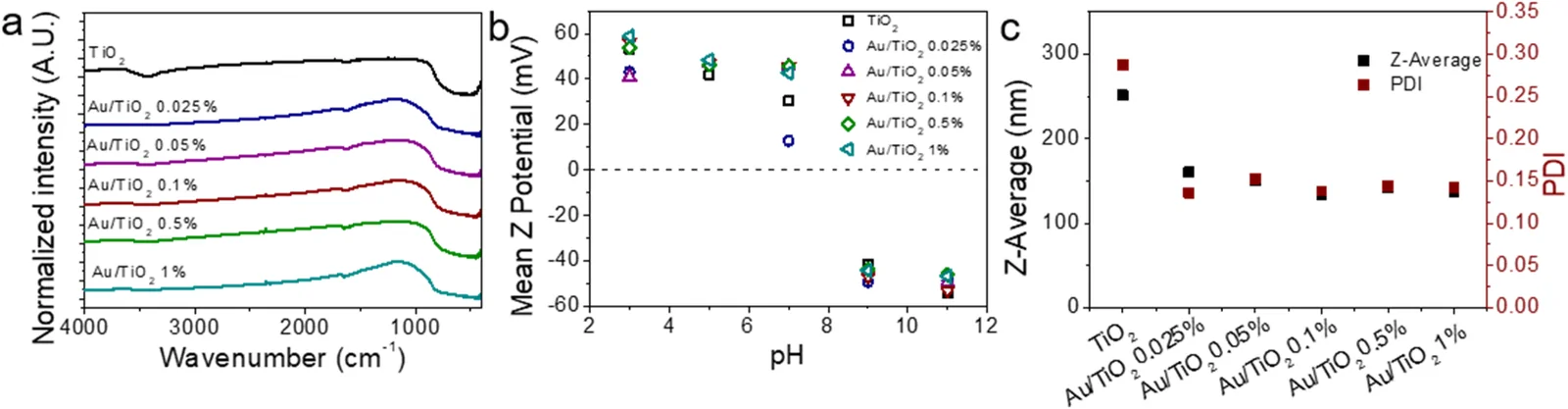
(a) FTIR spectra, (b) Z potential, and (c) DLS size measurements of Au/TiO_2_ synthesised at different Au loadings compared with pristine TiO_2_.

The stability of the synthesised nanocomposites was analysed by Z-potential measurements of aqueous dispersions at pH values ranging from 3 to 11 and compared with those for pristine TiO_2_. Results are shown in Fig. 5b. Au/TiO_2_ exhibits high stability at pH values below 7 and above 9, with values between |40| mV and |60| mV, forming a stable colloidal solution that carries positive charges at acidic pH and negative charges at basic pH.^50^ In all cases, the point of zero charge lies in the pH range of 7–8, which is higher than in other reports on Au/TiO_2_ synthesised by chemical methods.^17,30^

The average hydrodynamic size and polydisperse index (PDI) of Au/TiO_2_ and TiO_2_ are shown in Fig. 5c. Pristine TiO_2_ presents a hydrodynamic size of 251 ± 4 nm and a PDI value of 0.287, while the incorporation of Au leads to a decrease in the size of the nanocomposites to values between 160 and 130 nm and a PDI index of 0.153–0.136. This indicates that the nanocomposites tend to aggregate less and are less polydisperse than pristine TiO_2_, likely due to repulsive forces from Au on the surface of TiO_2_ nanoparticles.^51^

The spectra obtained by UV-Vis diffuse reflectance spectroscopy, shown in Fig. 6a, clearly distinguish pristine TiO_2_ from nanocomposites with different Au loadings. TiO_2_ reflects approximately 90% of incident light at wavelengths between 400 and 800 nm and less than 10% at wavelengths below 350 nm. After the addition of Au, the reflectance percentage in the 400–800 nm range decreases from 60% to 30% as the loading increases. Additionally, between 500 and 600 nm, the localised surface plasmon resonance (SPR) band of Au nanoparticles can be observed. Results are consistent with literature data indicating that Au-doped TiO_2_ exhibits a broader absorbance spectrum compared to pristine TiO_2_.^52–54^

**Fig. 6:**
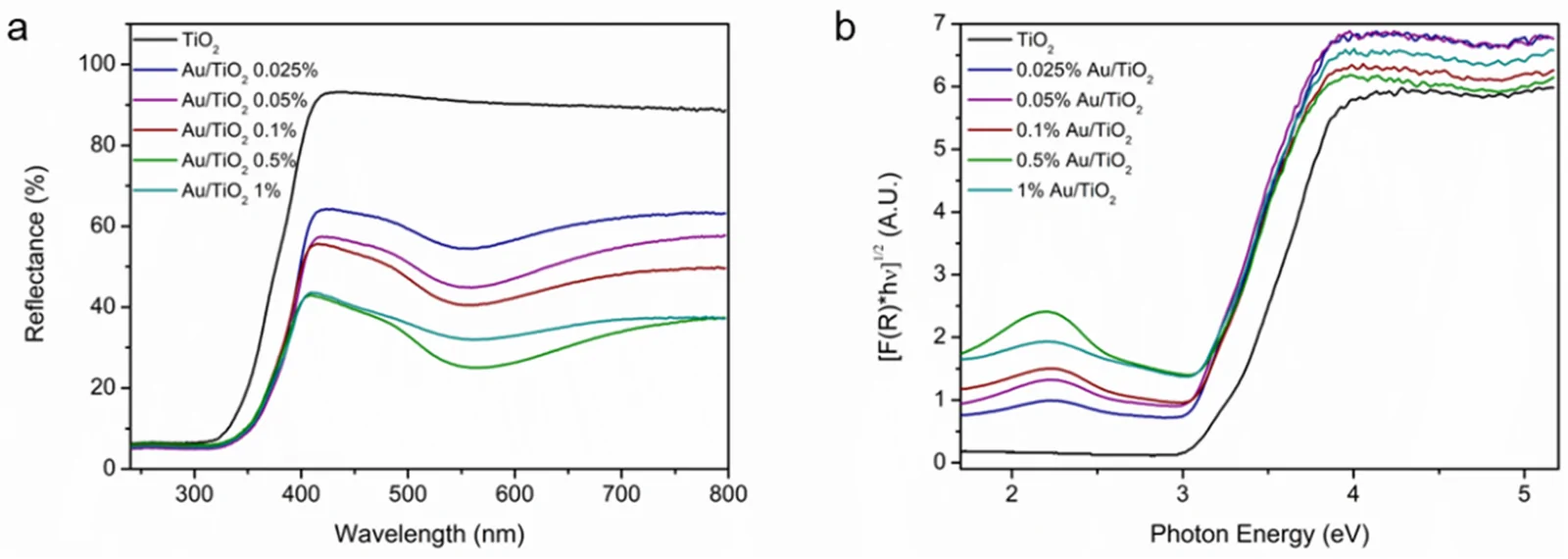
(a) UV-Vis reflectance spectra and (b) Kubelka–Munk plots for the estimation of the band gap energy of Au/TiO_2_ and comparison with pristine TiO_2_.

Table 2 presents the band gap energy (*E*_g_) values for the samples, calculated from Tauc plots (eqn (2)), as shown in Fig. 6b. A value of 3.05 eV was calculated for pristine TiO_2_, which is within the range of band gaps reported in the literature for TiO_2_ (2.96–3.39 eV).^17,55,56^ The calculated energy bandgap decreases in all nanocomposites, with values ranging from 2.94 eV at 0.025% loading to 2.87 eV at 1% gold loading. This trend is consistent with previous studies, despite differences in the synthesis methods. For instance, Saputra *et al.* synthesised Au/TiO_2_ nanocomposites *via* a green approach using *Pilea melastomoides* leaf and *Tamarindus indica* fruit extracts, reporting a band gap of 2.9 eV.^52^ Kanoun *et al.* employed a sol–gel-like method and observed a decrease in band gap from 3.09 eV for pure TiO_2_ to 2.82 eV and 2.78 eV for 0.5% and 2% Au loadings, respectively.^57^ These results support the red shift observed in the UV-assisted synthesis.

**Table 2:** Band gap energy estimated for the pristine TiO_2_ and the synthesised Au/TiO_2_ nanoparticles

Sample	*E*_g_ (eV)
TiO_2_	3.05 ± 0.02
0.025% Au/TiO_2_	2.94 ± 0.03
0.05% Au/TiO_2_	2.92 ± 0.02
0.1% Au/TiO_2_	2.92 ± 0.01
0.5% Au/TiO_2_	2.87 ± 0.01
1% Au/TiO_2_	2.87 ± 0.02

### 3.2 Detection of photogenerated hydroxyl singlet oxygen radicals

As previously indicated, when a photocatalyst such as TiO_2_ is irradiated with energy equal to or greater than its band gap, an e^−^/h^+^ pair is formed that can initiate redox reactions, generating ROS. Typically, photogenerated e^−^ reduce dissolved molecular oxygen (O_2_), resulting in the formation of a superoxide radical anion (O_2_˙−). This anion is then oxidised by h^+^ in the valence band, producing ^1^O_2_. Additionally, photogenerated h^+^ can generate ˙OH *via* reactions with surface hydroxyl groups (–OH) or H_2_O.^58,59^ In this context, the effect of the different Au loadings in the production of ˙OH and ^1^O_2_ has been determined, following previous reports on the quantification of ROS species generated by a photocatalyst^60,61^

The generation of ˙OH was quantified using the hydroxylation reaction of terephthalic acid. Fig. 7a shows the concentration of ˙OH produced for each nanocomposite at different UV irradiation times. The amount of singlet oxygen produced was quantified using the histidine test.^30^ The concentration of ^1^O_2_ obtained as a function of irradiation time for each Au/TiO_2_ sample is shown in Fig. 7b.

**Fig. 7:**
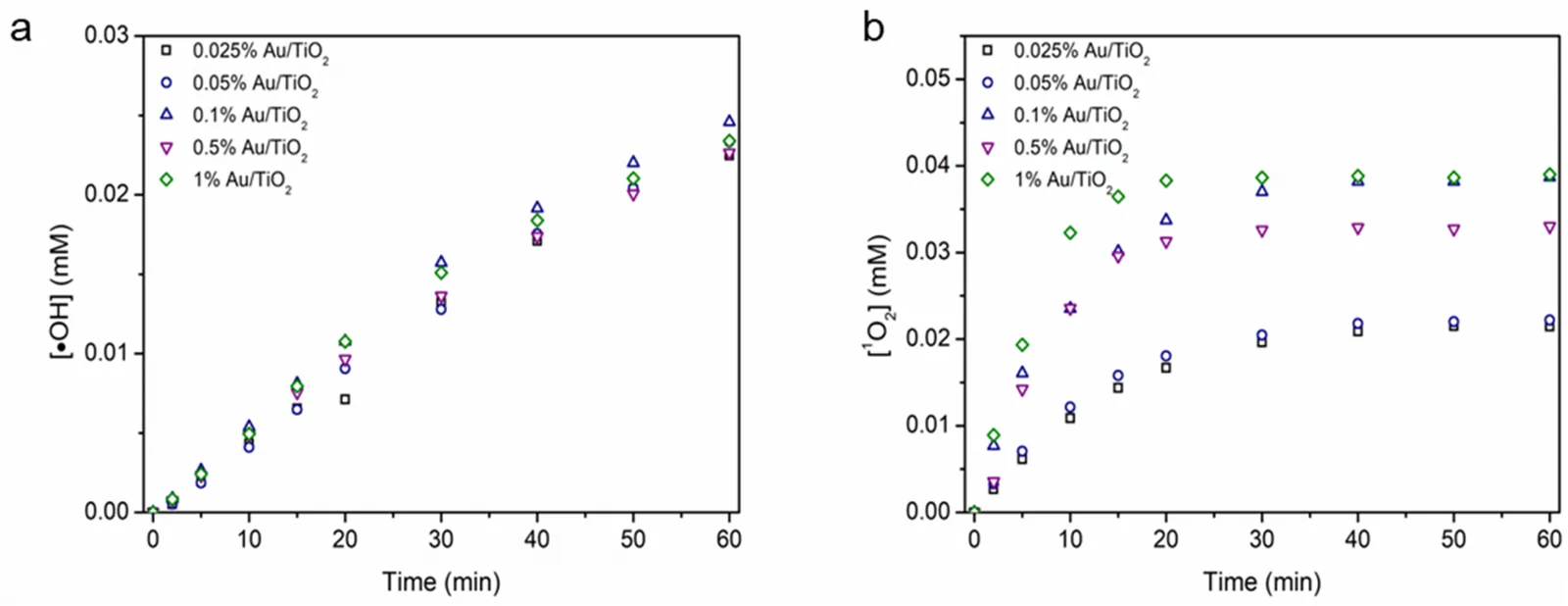
Concentration of (a) hydroxyl radicals and (b) singlet oxygen produced by Au/TiO_2_ with different Au loadings under UV irradiation.

An increase in hydroxyl radical production during the 60 min can be observed with almost no difference between the various gold loadings, as this species is formed through the oxidation of hydroxyl groups and water molecules by the h^+^ generated after the photoexcitation of the electron in the valence band to the conduction band, therefore being a mechanism only ascribed to TiO_2_.

In the production of singlet oxygen, a difference can be noted between the gold compositions in the samples. In all cases, an increase in the concentration of ^1^O_2_ is observed during the first min of irradiation, reaching a maximum at 20 min, after which it remains constant. The highest concentrations were obtained for the samples with 0.1 and 1% loadings of gold, almost double the concentration attained for the samples with 0.025 and 0.05%, since the formation of singlet involves the reduction of dissolved O_2_ by photogenerated electrons; the differences observed among the Au/TiO_2_ samples suggest that Au loading influences the availability and/or transfer of photogenerated charge carriers.^62^ However, direct evidence of the specific electron-transfer pathway cannot be established from the present measurements alone. The nanocomposite with 0.5% Au shows a lower concentration of ^1^O_2_ than that with 0.1%, but the results are, overall, consistent with band gap calculations, where the band gap of 2.94 eV for the nanocomposites with 0.025% Au was reduced to 2.87 eV when Au loading was 1%, and supplemented by literature that indicates photocatalytic efficiency of TiO_2_/Au is directly influenced by Au loading, particle size and synthesis method. These results confirm the ability of the Au/TiO_2_ nanocomposites to generate detectable ROS under irradiation. However, their relative contribution to the degradation of the investigated CECs cannot be established solely from the ROS-generation assays. Therefore, the observed differences in ROS generation are considered indicative of changes in the photocatalytic behaviour induced by Au incorporation, rather than as definitive evidence of a dominant degradation pathway.

### 3.3 Photocatalytic degradation evaluation

After assessing the nanocomposites' ability to generate ROS, their photocatalytic activity for the degradation of the antibiotic CIP was evaluated under UV and simulated sunlight, using a CIP concentration of 5 ppm to compare with previous studies.^17,30^ This CIP concentration remained stable and was not degraded under UV and simulated sunlight radiation (photolysis) without the presence of a photocatalyst. The photolysis spectra for these assays are shown in the supplementary material (Fig. S1a and b).

Fig. 8a and Table 3 present the results of CIP degradation under UV radiation for Au/TiO_2_ with varying gold loadings, compared with pristine TiO_2_. Although the adsorption percentage of CIP in the Au/TiO_2_ samples decreases compared with TiO_2_, the results show an enhanced degradation percentage, with values increasing from 85% for pristine TiO_2_ to 94% for 1% Au/TiO_2._

**Fig. 8:**
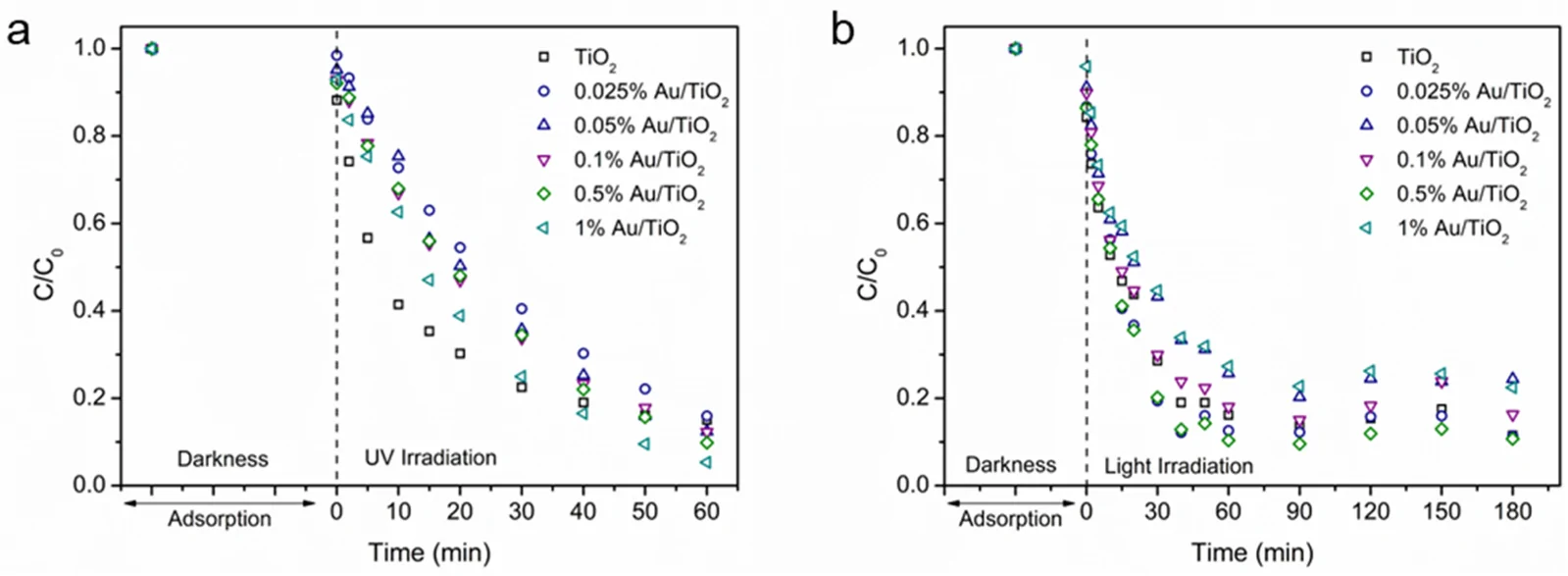
Photocatalytic degradation of CIP using Au/TiO_2_ with different Au concentrations compared with bare TiO_2_ nanoparticles under (a) UV irradiation, (b) simulated sunlight.

**Table 3:** CIP percentage of adsorption, degradation efficiency, and corresponding apparent first-order reaction rate constants (*k*) under 60 min of UV irradiation, 60 min and 180 min of simulated sunlight irradiation for TiO_2_ and Au/TiO_2_ at different gold loadings

Sample	UV radiation				Simulated sunlight		
	Au wt%	% Adsorption	% Degradation (60 min)	*k* (min^−1^)	% Degradation (60 min)	% Degradation (180 min)	*k* (min^−1^)
TiO_2_	0	12%	85%	0.045 ± 0.005	83%	88%	0.033 ± 0.002
Au/TiO_2_	0.025	2%	84%	0.030 ± 0.004	87%	89%	0.048 ± 0.002
	0.05	5%	88%	0.034 ± 0.002	74%	75%	0.024 ± 0.002
	0.1	7%	88%	0.034 ± 0.003	82%	84%	0.035 ± 0.001
	0.5	8%	90%	0.033 ± 0.006	90%	90%	0.047 ± 0.001
	1	7%	94%	0.044 ± 0.008	73%	77%	0.024 ± 0.003

Interestingly, despite improved degradation efficiency, there is no increase in the apparent reaction rate; *k* values for TiO_2_ and 1% Au/TiO_2_ are similar, at 0.045 and 0.044 min^−1^, respectively. In contrast, the Au/TiO_2_ nanocomposites with 0.025%, 0.05%, 0.1%, and 0.5% Au exhibit values ranging from 0.030 to 0.034 min^−1^. There have been discrepancies in the literature regarding the reported enhanced photocatalytic activity of Au/TiO_2_ under UV irradiation, with some studies suggesting that gold nanoparticles only improve degradation efficiency in the visible spectrum due to plasmonic absorption.^63^ Other works, however, report enhanced photocatalysis that increases with Au loading due to charge separation, as the gold nanoparticles trap the excited electrons of TiO_2_. ^17^ As observed in the present photocatalytic assays, in the early stages of degradation, the adsorption equilibrium may limit efficiency, and apparent reaction rates may be lower or similar to those of pristine TiO_2_, despite higher degradation efficiencies.

The results obtained from the assay under simulated sunlight irradiation are presented in Fig. 8b and Table 3.

For pristine TiO_2_, the degradation efficiency after 60 min is 83%, similar to the 85% observed under UV irradiation for the same duration, and longer irradiation times do not improve efficiency significantly (88%). This was expected since TiO_2_ primarily absorbs the UV portion of the simulated sunlight spectrum.

The addition of Au nanoparticles increases degradation efficiency, as observed in samples with 0.025% and 0.5% Au content, reaching 89% and 90%, respectively, after 180 min. Notably, the 0.5% Au/TiO_2_ sample achieves 90% CIP degradation after just 60 min of simulated sunlight irradiation, with an apparent reaction rate constant of 0.047 min^−1^. However, samples with Au contents of 0.05%, 0.1%, and 1% did not show improved degradation efficiency, exhibiting lower degradation efficiency than pristine TiO_2_.

When doping TiO_2_ with metal nanoparticles, a compromise between the beneficial and detrimental effects of metal incorporation is necessary. Gold nanoparticles can act as electron-trapping sites at the TiO_2_ interface, potentially favouring charge separation by accepting photogenerated electrons from TiO_2_ and thereby decreasing the probability of electron–hole recombination.^63^ In the present study, the enhanced photocatalytic performance observed at intermediate Au loadings is consistent with this contribution; however, direct evidence of reduced charge-carrier recombination was not obtained. Furthermore, the LSPR of Au can enhance the photocatalytic response by extending the optical absorption of TiO_2_ towards longer wavelengths, increasing light scattering, and potentially promoting interfacial charge-transfer processes.^64,65^ However, increasing the Au content beyond an optimum value can also have detrimental effects, including partial coverage of the TiO_2_ surface, shielding of active sites and attenuation of light reaching the semiconductor, while higher metal loading may additionally favour the formation of larger Au domains or local aggregation.^17,62,63^ These effects can reduce the effective Au/TiO_2_ interfacial area and the accessibility of TiO_2_ active sites for pollutant adsorption and photocatalytic reactions.

This balance is consistent with the observed dependence of photocatalytic activity on Au loading. While Au contents of 0.025, 0.05 and 0.1 wt% were insufficient to produce a clear improvement over pristine TiO_2_, increasing the loading to 0.5 wt% resulted in the highest photocatalytic activity. Further increasing the Au content to 1 wt% led to a decrease in degradation efficiency, suggesting that the additional Au does not necessarily translate into a proportional enhancement of photocatalytic activity. Interestingly, although the 1 wt% Au/TiO_2_ sample showed enhanced ^1^O_2_ generation, it did not exhibit the highest photocatalytic activity, indicating that ROS generation alone cannot account for the observed optimum at 0.5 wt% Au. Therefore, the superior performance of the 0.5 wt% material likely results from a balance between the beneficial effects of Au incorporation and the possible adverse effects associated with excessive Au coverage. These interpretations should be considered as possible contributing factors, since direct charge-carrier measurements were not obtained for all Au loadings.

Therefore, based on these results, 0.5% Au/TiO_2_ was selected for further analysis of the degradation efficiency of other pharmaceutical compounds under UV and simulated sunlight. For comparison, the same amount of photocatalyst was employed in each experiment. However, the contaminant concentration was adjusted to monitor degradation using UV-Vis spectroscopy. The pharmaceuticals employed were Chloroquine phosphate (CLQ, 30 ppm), Diclofenac sodium (DIC, 30 ppm), Paracetamol (PAR, 15 ppm) and Fluconazole (FLU, 200 ppm). The stability of the drugs under UV and simulated sunlight irradiation was assessed, and the photolysis spectra are presented in Fig. S1, showing no degradation for any of them at the tested times. Fig. 9 and Table 4 summarise the observed degradation efficiencies.

**Fig. 9:**
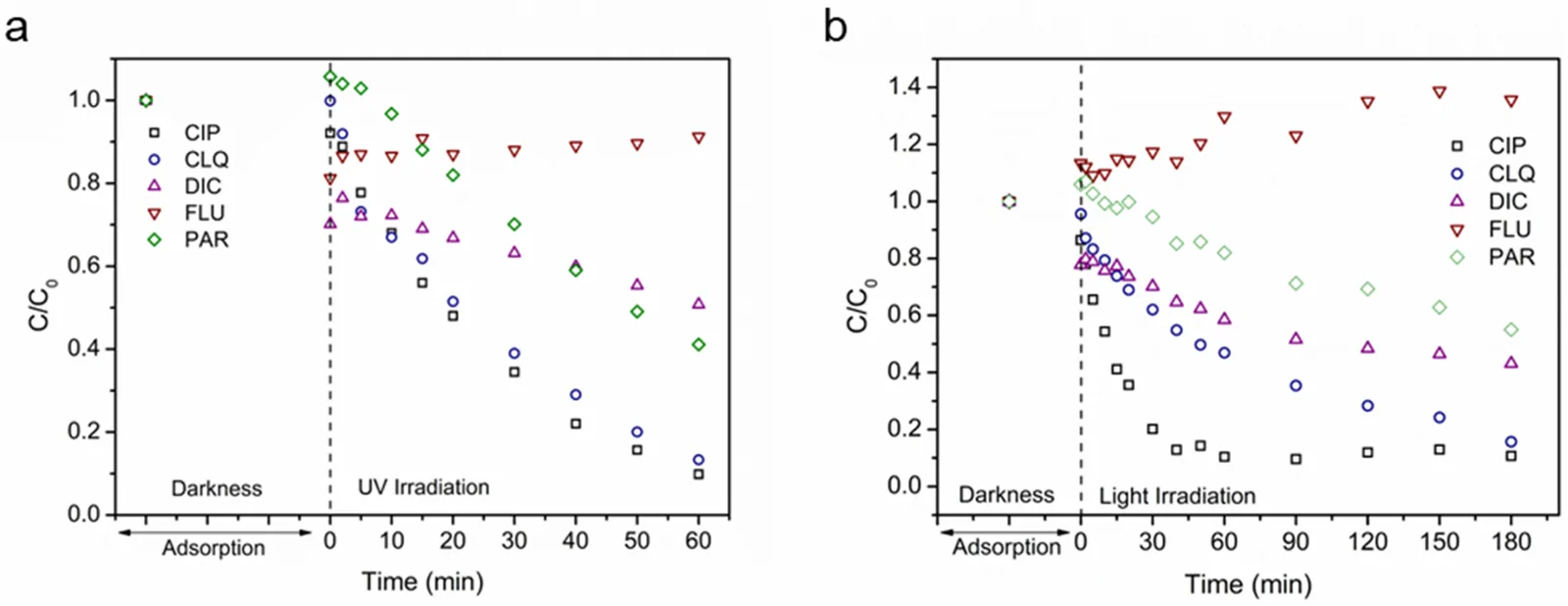
Photocatalytic degradation of CIP (5 ppm), CLQ (30 ppm), DIC (30 ppm), PAR (15 ppm) and FLU (200 ppm) using 0.5% Au/TiO_2_ under (a) UV irradiation, (b) simulated sunlight.

**Table 4:** Percentage of adsorption, degradation efficiency and corresponding apparent reaction rate constants (*k*) under 60 min of UV irradiation, 60 min and 180 min of simulated sunlight irradiation for the different pharmaceuticals with 0.5% Au/TiO_2_

Sample	UV radiation			Simulated sunlight		
	% Adsorption	% Degradation (60 min)	*k* (min^−1^)	% Degradation (60 min)	% Degradation (180 min)	*k* (min^−1^)
CIP	8%	90%	0.033 ± 0.0006	90%	90%	0.047 ± 0.001
CLQ	0.1%	87%	0.148 ± 0.002	51%	84%	0.067 ± 0.001
DIC	29%	27%	0.005 ± 0.001	25%	45%	0.004 ± 0.01
FLU	—	—	—	—	—	—
PAR	0%	61%	0.014 ± 0.0007	22%	48%	0.004 ± 0.001

As explained in Section 2.5, each pharmaceutical's chemical structure and polarity influence adsorption affinity and interaction with ROS and the irradiation source.

Despite the cationic nature of CIP at pH 3, which should promote repulsion with the positively charged Au/TiO_2_ nanocomposites (Fig. 5b), a moderate adsorption (8%) was observed, which can be attributed to non-electrostatic interactions (π–π stacking and hydrogen bonding). The high degradation efficiencies (90%) under both UV radiation and simulated sunlight can be attributed to the interaction between its electron-donating groups (piperazine nitrogen, carboxylic acid, and quinoline ring) and the generated ROS.^66,67^ The increase in rate constants from 0.033 (UV) to 0.047 (simulated sunlight) also reflects the effective plasmonic activation of Au/TiO_2_ under visible light, explaining the plateau degradation after 60 min of irradiation.

CLQ showed low adsorption affinity (0.1%) due to its dicationic form at pH 3. However, its degradation was still efficient under UV radiation (87%, *k* = 0.148 min^−1^) and simulated sunlight (84%, *k* = 0.067 min^−1^), owing to the presence of quinoline rings. CLQ is more photoreactive than the other pharmaceuticals under UV radiation, which explains its faster degradation kinetics.^68–70^

DIC, despite strong adsorption (29%) due to its neutral charge in these experimental conditions and other non-electrostatic interactions, exhibited poor degradation under both UV (27%, *k* = 0.005 min^−1^) and simulated sunlight (45% at 180 minutes, *k* = 0.004 min^−1^), since its electron-deficient, chlorinated aromatic rings are not only resistant to oxidation but may also reduce ROS, particularly hydroxyl radicals, thus impeding the degradation process ^71^.

PAR had no measurable adsorption because of its highly polar molecular structure and lack of functional groups that could strongly bind to TiO_2_, which consequently hinders degradation under UV (61%, *k* = 0.014 min^−1^) and simulated sunlight (48% at 180 minutes, *k* = 0.004 min^−1^) radiation, despite interactions between ROS and its aromatic phenolic hydroxyl group.^72,73^

FLU was neither adsorbed nor degraded by the photocatalyst due to electrostatic repulsions between its cationic molecules and the positively charged Au/TiO_2_ nanocomposites that prevent interactions with ROS, as well as the presence of fluorinated aromatic systems and triazole rings in its structure, which are stable against oxidation.^74^

It should be noted that the decrease in the UV-Vis absorption of the pharmaceuticals reflects the disappearance of the parent compounds but does not provide direct information on their mineralisation or on the possible formation of transformation products.

Photocatalytic efficiency is influenced by the chemical structure of the target contaminant and the physical and chemical characteristics of the photocatalyst, which are determined by the synthesis method. Table 5 compares the performance of Au/TiO_2_ nanocomposites synthesised using three approaches: UV-driven photodeposition (this study), conventional co-precipitation, and a green co-precipitation method employing a flavonoid as a reducing agent. The photocatalytic activity was evaluated against ciprofloxacin (CIP, 5 mg L^−1^) under UV and simulated solar irradiation. It is important to note that degradation efficiency is also affected by other experimental conditions, such as radiation intensity, irradiation time, and the concentrations of the photocatalyst, Au load, and contaminant.

**Table 5:** Comparison of the photocatalytic efficiency obtained in the present work with previous studies using Au/TiO_2_ nanocomposites under UV radiation and simulated sunlight against CIP

Synthesis method	Au concentration	Radiation	Irradiation time (min)	% Degradation	Reference
Pristine P25 TiO_2_	0 wt%	UV	60	85	Present work
		Simulated sunlight	180	88	
UV-driven photodeposition	0.5 wt%	UV	60	90	
		Simulated sunlight	180	90	
Conventional co-precipitation	0.05 wt%	UV	30	91	17
		Simulated sunlight	180	49	
Green co-precipitation	0.1 wt%	UV	60	74	20
		Simulated sunlight	180	75	

The conventional co-precipitation method of functionalizing pristine TiO_2_ with Au nanoparticles appears to increase the photocatalytic activity against CIP from 85% to 91% under UV radiation, despite the shorter irradiation time. This means that although efficiency is highly dependent on TiO_2_ ROS production under high-energy radiation, the Au nanoparticles can capture some of the photoexcited electrons, thereby reducing the rate of electron–hole pair recombination. Under simulated sunlight, however, efficiency dropped to 49% after 180 min, probably because the low Au content (0.05 wt%) limits visible-light absorption. The green co-precipitation method, utilising a flavonoid as a reducing agent to incorporate a higher Au load (0.1%), yielded adequate degradation efficiencies (74% under UV and 75% under simulated sunlight). Comparing these results to the efficiencies of 90% that the nanocomposites obtained in this work achieved under both UV and simulated sunlight radiation, it can be concluded that the newly developed UV-driven photodeposition method might yield smaller or more uniformly distributed Au particles (Fig. 2), increasing the number of active sites and improving charge separation, which consequently enhances photocatalytic efficiency across a broader radiation spectrum, suitable for UV or solar-driven applications.

To further contextualise the photocatalytic performance of the present Au/TiO_2_ nanocomposites, a broader comparison with representative TiO_2_-based photocatalysts reported for the degradation of the other pharmaceutical contaminants investigated in this work is provided in Table 6. Priority was given to Au/TiO_2_ systems, when available, while representative TiO_2_-based photocatalysts modified with other noble metals or semiconductor heterojunctions were considered when directly comparable Au/TiO_2_ systems were not identified. The comparison includes the main experimental parameters reported in the literature, such as catalyst dosage, irradiation source, irradiation time, degradation efficiency and apparent kinetic constants, when available. Because photocatalytic performance is strongly dependent on the experimental conditions employed, the values reported in Table 6 should be considered as a contextual comparison rather than a direct ranking of photocatalysts.

**Table 6:** Comparison of the photocatalytic degradation of the investigated pharmaceutical contaminants with representative TiO_2_-based photocatalytic systems reported in the literature

Pharmaceutical	Photocatalyst	Dose	Radiation	Irradiation time (min)	% Degradation	Reference
CLQ	Au/TiO_2_	1 mg mL^−1^	UV	60	87%	Present work
			Simulated sunlight	180	84%	
	Au/TiO_2_	1 mg mL^−1^	UV	60	50%	30
			Xenon	240	70%	
	TiO_2_@FeS_2_	250 mg L^−1^	UV-C	120	92%	75
	Bi_2_O_3_-TiO_2_/PAC	250 mg L^−1^	Xenon	30	95%	76
	TiO_2_	1 mg mL^−1^	UV	60	83%	77
DIC	Au/TiO_2_	1 mg mL^−1^	UV	60	27%	Present work
			Simulated sunlight	180	45%	
	Au/TiO_2_	1 mg mL^−1^	Xenon	240	53%	30
	TiO_2_/SiO_2_/Cu	0.5 g L^−1^	Solar simulator	120	98%	78
	MoS_2_/TiO_2_/Fe_3_O_4_	0.2 g L^−1^	Xenon	6	99.6%	79
	Au/TiO_2_	200 ppm	UV	300	47	80
	TiO_2_	0.6 g L^−1^	Simulated sunlight	180	20%	81
	PAN-MWCNT/TiO_2_	1 g L^−1^	UV	90	91%	82
			Visible light	360	56%	
	PAN-MWCNT/TiO_2_-NH_2_		UV	90	90%	
			Visible light	360	67%	
PAR	Au/TiO_2_	1 mg mL^−1^	UV	60	61%	Present work
			Simulated sunlight	180	48%	
	Au/TiO_2_	1 mg mL^−1^	UV	60	23%	30
			Xenon	240	61%	
	Au/TiO_2_	100 mg L^−1^	Xenon	240	90%	83
	Au/TiO_2_	5 mg L^−1^	UV	60	n.d.	84
	Au/TiO_2_	n.d.	UV	30	85.7%	85
	Au/TiO_2_		Simulated sunlight	180	68.8%	
	Au/TiO_2_/ZnO	0.3 mg mL^−1^	Visible light	50	76.7%	86
	Au/TiO_2_	0.5 g L^−1^	UV	180	n.d.	87
	Au/TiO_2_	1 g L^−1^	Solar	180	99%	47
	Au/TiO_2_	0.3 mg mL^−1^	UV	n.d.	n.d.	88
			Visible light	n.d.	n.d.	

## 4 Conclusions

A UV-driven one-pot photodeposition method was developed to functionalise TiO_2_ with Au nanoparticles without an additional chemical reducing agent. The synthesis preserved the crystalline structure and morphology of TiO_2_, while STEM-EDX and HRTEM analyses confirmed the presence and crystalline nature of the deposited Au. The incorporation of Au modified the optical properties of TiO_2_, increasing visible-light absorption and decreasing the estimated band gap with increasing Au loading. The Au/TiO_2_ nanocomposites also showed a reduced hydrodynamic size and polydispersity compared with pristine TiO_2_. The materials generated reactive oxygen species under UV irradiation, with •OH production remaining relatively consistent across different Au loadings, whereas ^1^O_2_ generation depended on the Au content. Photocatalytic experiments demonstrated a modest improvement in CIP degradation under UV irradiation and a more pronounced enhancement under simulated solar radiation, with 0.5 wt% Au/TiO_2_ achieving 90% degradation after 60 min compared with 83% for pristine TiO_2_. Although these improvements may be associated with modified interfacial charge–transfer processes and, under simulated sunlight, with the SPR of Au, the specific contribution of charge separation and electron-transfer processes cannot be directly established from the present measurements.

The degradation of CLQ, DIC, PAR and FLU further demonstrated that photocatalytic performance depends on the chemical characteristics of the target contaminant. Overall, the proposed aqueous photodeposition route provides a simple alternative for preparing Au/TiO_2_ nanocomposites without an additional chemical reducing agent, while the use of low Au loadings and the identification of 0.5 wt% as the optimal loading under the conditions investigated contributes to the efficient use of the noble metal. Nevertheless, catalyst recyclability, long-term stability, Au leaching, and performance in complex water matrices were not evaluated in this study. Future work should address these aspects, including the development of immobilised Au/TiO_2_ configurations to facilitate catalyst recovery and reuse, as well as the evaluation of potential nanoparticle release and ecotoxicological effects. Further assessment under natural sunlight and in real water matrices, together with energy and techno-economic analyses, will also be required to establish the overall sustainability and scalability of the proposed photocatalytic technology.

## Conflicts of interest

There are no conflicts to declare.

## Supplementary Material

RA-016-D6RA06617H-s001

## Data Availability

The data supporting the findings of this study are available within the article and its supplementary information (SI). Supplementary information is available. See DOI: https://doi.org/10.1039/d6ra06617h.
