# Supplementary material for: Green photodeposition of Au/TiO2 nanocomposites for wide-range photocatalytic degradation of pharmaceuticals
Source: RSC Adv. 2026 Oct 14;16(47):62917–31. doi: 10.1039/d6ra06617h (PMC13664701; doi:10.1039/d6ra06617h)
Supplement: Supplementary file 1 [file RA-016-D6RA06617H-s001.pdf]

This journal is © The Royal Society of Chemistry

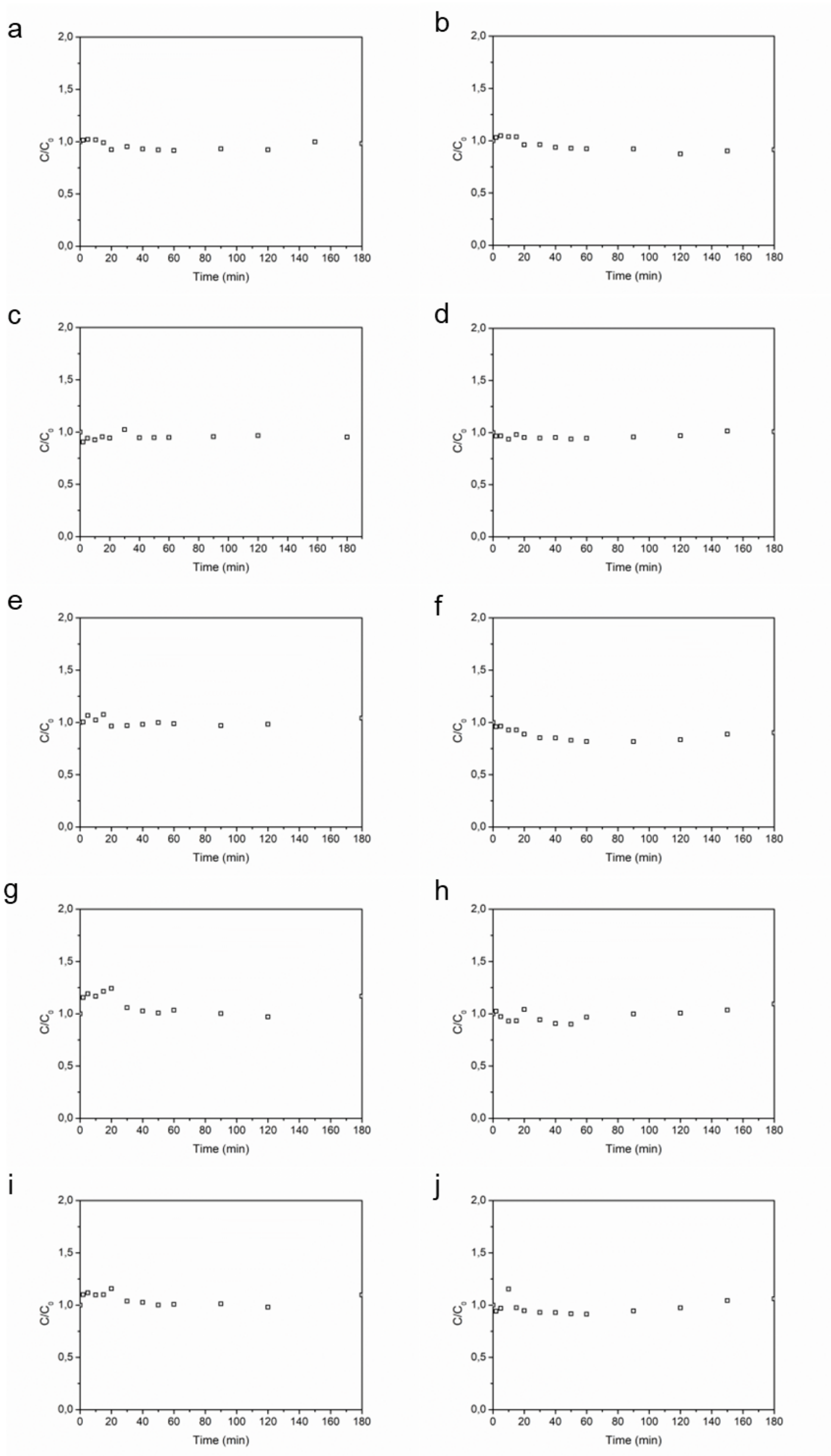

Figure S1 - Photolysis of a and b Ciprofloxacin, c and d Chloroquine phosphate, e and f Diclofenac sodium, g and h Fluconazole, I and j Paracetamol under UV and simulate sunlight, respectively.
